# Assessment of solutions from the consistently linearized eigenproblem by means of finite difference approximations

**DOI:** 10.1016/j.compstruc.2015.01.016

**Published:** 2015-04-15

**Authors:** X. Jia, H.A. Mang

**Affiliations:** a Institute for Mechanics of Materials and Structures, Vienna University of Technology, Karlsplatz 13/202, 1040 Vienna, Austria; b National RPGE Chair Professor, Tongji University, Siping Road 1239, Shanghai, China

**Keywords:** Buckling, Consistently linearized eigenproblem, Co-rotational beam element, Derivatives of the tangent stiffness matrix, Load-based and displacement-based finite difference approximations

## Abstract

The consistently linearized eigenproblem (CLE) plays an important role in stability analysis of structures. Solution of the CLE requires computation of the tangent stiffness matrix ${\overset{∼}{K}}_{T}$ and of its first derivative with respect to a dimensionless load parameter *λ*, denoted as ${\overset{˙}{\overset{∼}{K}}}_{T}$. In this paper, three approaches of computation of ${\overset{˙}{\overset{∼}{K}}}_{T}$ are discussed. They are based on (a) an analytical expression for the derivative of the element tangent stiffness matrix ${\overset{∼}{K}}_{T}^{e}$, (b) a load-based finite difference approximation (LBFDA), and (c) a displacement-based finite difference approximation (DBFDA). The convergence rate, the accuracy, and the computing time of the LBFDA and the DBFDA are compared, using the analytical solution as the benchmark result. The numerical investigation consists of the analysis of a circular arch subjected to a vertical point load at the vertex, and of a thrust-line arch under a uniformly distributed load. The main conclusion drawn from this work is that the DBFDA is superior to the LBFDA.

## Highlights

• The consistently linearized eigenproblem (CLE) is solved.
• Derivative of the tangent stiffness matrix w.r.t. load factor is derived.
• The aforementioned derivative is also approximated numerically.
• The displacement-based approximation is effective and efficient.

## 1 Introduction

Buckling is one of the most important causes of loss of the integrity of structures. Hence, the investigation of this phenomenon is very important in structural analysis and design. The so-called consistently linearized eigenproblem (CLE), originally proposed in [1], [2], was initially used for *ab initio* estimates of stability limits by means of the Finite Element Method (FEM) [3]. Herein, *ab initio* means “without incremental analysis”. Compared to other modes of accompanying linear eigenvalue analysis, as proposed e.g. in [4], [5], for specific problems, such as, for example the von Mises truss subjected to a point load at the vertex, the CLE provides a better estimation of the buckling load (see Fig. 2.19 in [2]). Later on, Mang and collaborators employed the CLE for assessing the initial postbuckling behavior of elastic structures [6], [7], [8]. The CLE also plays a role in the energetical classification of limiting cases of loss of stability such as lateral torsional buckling [9].

The mathematical formulation of the CLE reads as

(1) $$[{\overset{∼}{K}}_{T}+(\lambda_{j}^{*}-\lambda ){\overset{˙}{\overset{∼}{K}}}_{T}]\cdot v_{j}^{*}=0\text{,}\;j=1\text{,}2\text{,}3\text{,}\ldots \text{,}N\text{,}$$

where ${\overset{∼}{K}}_{T}$ denotes the tangent stiffness matrix of a structure in the frame of FEM, evaluated along the primary path;

(2) $${\overset{˙}{\overset{∼}{K}}}_{T}≔\frac{d{\overset{∼}{K}}_{T}}{d\lambda }\text{,}$$

where *λ* stands for a dimensionless load factor; $\left(\lambda_{j}^{*}-\lambda \text{,}\;v_{j}^{*}\right)$ is the *j-th* eigenpair, with

(3) $$v_{j}^{*}\cdot v_{j}^{*}=1\text{.}$$

The relevant eigenpair is the one that is associated with loss of stability, characterized by the semi-positive definiteness of ${\overset{∼}{K}}_{T}$. It is given as

(4) $$(\lambda_{1}^{*}-\lambda \text{,}\;v_{1}^{*})\text{.}$$

To solve the CLE, ${\overset{˙}{\overset{∼}{K}}}_{T}$ needs to be computed in addition to ${\overset{∼}{K}}_{T}$. Proposing an efficient strategy for calculation of ${\overset{˙}{\overset{∼}{K}}}_{T}$ is the main task of this work.

Three approaches for calculation of ${\overset{˙}{\overset{∼}{K}}}_{T}$ will be discussed. The first one is based on an analytical expression for the first derivative of the tangent stiffness matrix of element $e\text{,}{\overset{∼}{K}}_{T}^{e}$, with respect to *λ* for the special case of a co-rotational beam element. The second one is a finite difference approach, herein referred to as load-based finite difference approximation (LBFDA); the third one is also a finite difference approach, herein designated as displacement-based finite difference approximation (DBFDA). These two designations reflect the specific character of the two finite difference approximations. Solutions of the CLE, based on the first approach, are considered as the benchmark results. Results obtained by means of the LBFDA and the DBFDA will be compared with corresponding results obtained with the help of the analytical method. The comparison involves the convergence rate, the accuracy, and the computing time.

This paper is organized as follows: in Section 2, basic mathematical properties of the CLE will be presented. Section 3 is devoted to the derivation of an analytical expression for ${\overset{˙}{\overset{∼}{K}}}_{T}$ for a co-rotational beam element. In Section 4, the two finite difference approaches will be delineated, including consideration of programming aspects. In Section 5, the theoretical findings will be verified in the frame of a numerical investigation. In particular, a circular arch subjected to a vertical point load at the vertex, and a thrust-line arch subjected to a uniformly distributed load will be investigated with special emphasis on loss of stability. Conclusions from this investigation will be drawn in Section 6.

## 2 Basic mathematical properties of the consistently linearized eigenproblem

**Fig. 1:**
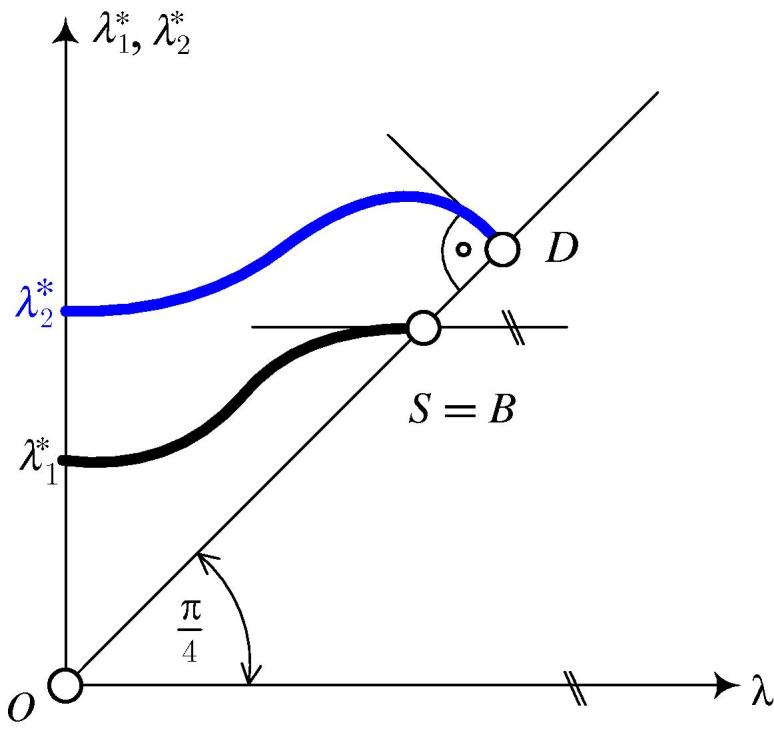
$\lambda_{1}^{*}-\lambda$ and $\lambda_{2}^{*}-\lambda$ diagram referring to bifurcation buckling and snap-through buckling, respectively.

The $\lambda_{1}^{*}$-*λ* ($\lambda_{2}^{*}$-*λ*) diagram in Fig. 1 is related to the eigenvalue function $\lambda_{1}^{*}(\lambda )-\lambda$ ($\lambda_{2}^{*}(\lambda )-\lambda$). The $\lambda_{1}^{*}$-*λ* curve refers to bifurcation buckling. S=B denotes a stability limit of the form of a bifurcation point. At this point [6],

(5) $$\lambda_{1}^{*}=\lambda \text{,}\;d\lambda \;\neq \;0\text{,}\;\mathrm{and}\;{\overset{˙}{\lambda }}_{1}^{*}=0\text{.}$$

The $\lambda_{2}^{*}-\lambda$ diagram refers to snap-through which, because of $\lambda_{D}>\lambda_{B}$, is not relevant in the given case. At point *D* [6], denoting the snap-through point,

(6) $$\lambda_{1}^{*}=\lambda \text{,}\;d\lambda =0\text{,}\;\mathrm{and}\;{\overset{˙}{\lambda }}_{1}^{*}=-1\text{.}$$

Writing (1) for the first eigenpair and differentiating the obtained relation with respect to *λ* gives

(7) $$[{\overset{˙}{\lambda }}_{1}^{*}{\overset{˙}{\overset{∼}{K}}}_{T}+(\lambda_{1}^{*}-\lambda ){\overset{¨}{\overset{∼}{K}}}_{T}]\cdot v_{1}^{*}+[{\overset{∼}{K}}_{T}+(\lambda_{1}^{*}-\lambda ){\overset{˙}{\overset{∼}{K}}}_{T}]\cdot {\overset{˙}{v}}_{1}^{*}=0\text{.}$$

where

(8) $${\overset{˙}{v}}_{1}^{*}=\overset{N}{\underset{j=2}{\sum }}c_{1j}v_{j}^{*}\text{.}$$

(7) holds for buckling from a general stress state, and $c_{1j}$ denotes the contribution of the eigenvector $v_{j}^{*}$ to ${\overset{˙}{v}}_{1}^{*}$. For buckling from a membrane stress state

(9) $${\overset{˙}{v}}_{1}^{*}=0\text{,}$$

resulting in

(10) $$[{\overset{˙}{\lambda }}_{1}^{*}{\overset{˙}{\overset{∼}{K}}}_{T}+(\lambda_{1}^{*}-\lambda ){\overset{¨}{\overset{∼}{K}}}_{T}]\cdot v_{1}^{*}=0\text{.}$$

In Section 5.2, this special case will be verified numerically.

## 3 Analytical expression for ${\overset{˙}{\overset{∼}{K}}}_{T}$ based on a co-rotational beam element

For a static, conservative system with *N* degrees of freedom (DOF), the infinitesimally incremental form of the equilibrium equations can be written as

(11) $${\overset{∼}{K}}_{T}\cdot \overset{˙}{q}=\overset{¯}{P}\text{,}$$

where $\overset{¯}{P}$ represents the vector of reference node forces, and

(12) $$\overset{˙}{q}=\frac{dq}{d\lambda }\text{,}$$

with q denoting the vector of node displacements. In the frame of the FEM, ${\overset{∼}{K}}_{T}$ is obtained by assembling the element tangent stiffness matrices ${\overset{∼}{K}}_{T}^{e}\text{,}e=1\text{,}2\text{,}\ldots \text{,}M$, i.e.,

(13) $${\overset{∼}{K}}_{T}=\overset{M}{\underset{e=1}{\sum }}A^{\mathrm{eT}}\cdot {\overset{∼}{K}}_{T}^{e}\cdot A^{e}$$

where *M* denotes the number of elements, and $A^{e}$ is the connectivity matrix of element *e*. In (13), ${\overset{∼}{K}}_{T}^{e}$ is a n×n matrix, with *n* standing for the number of DOF of an element, and $A^{e}$ is a n×N matrix. Since $A^{e}$ is constant,

(14) $${\overset{˙}{A}}^{e}=0\text{.}$$

Differentiation of (13) with respect to *λ* and consideration of (14) yields

(15) $${\overset{˙}{\overset{∼}{K}}}_{T}=\overset{M}{\underset{e=1}{\sum }}A^{\mathrm{eT}}\cdot {\overset{˙}{\overset{∼}{K}}}_{T}^{e}\cdot A^{e}\text{.}$$

Eq. (15) shows that ${\overset{˙}{\overset{∼}{K}}}_{T}$ can be obtained by assembling ${\overset{˙}{\overset{∼}{K}}}_{T}^{e}$. Hence, differentiation of the N×N matrix ${\overset{∼}{K}}_{T}$ is reduced to differentiation of the n×n matrix ${\overset{∼}{K}}_{T}^{e}$. Because of N≫n, this results in a very significant reduction of the analytical work.

**Fig. 2:**
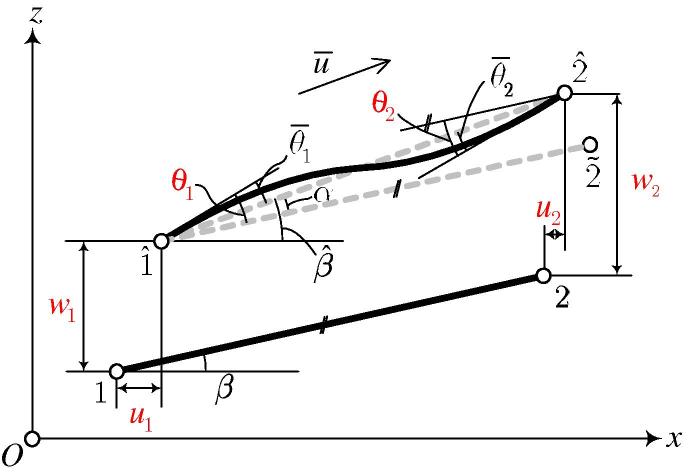
Kinematics of a two-dimensional co-rotational beam.

Because of its simplicity, the co-rotational approach is widely used in nonlinear finite element analysis [10], [11], [12]. Herein, a two-dimensional co-rotational beam element, based on the Euler–Bernoulli assumptions, is developed. The displacement of the element is decomposed into the rigid-body part and the deformational part, as shown in Fig. 2. The rigid-body displacement includes two parts, a translation (from $\overset{‾}{12}$ to $\overset{‾}{\overset{ˆ}{1}\overset{\sim }{2}}$) and a rotation (from $\overset{‾}{\overset{ˆ}{1}\overset{\sim }{2}}$ to $\overset{‾}{\overset{ˆ}{1}\overset{ˆ}{2}}$, described by the angle *α*). In the global coordinate system (x,z) the displacement vector is given as

(16) $$q^{e}={\left\lfloor u_{1}\text{,}\;w_{1}\text{,}\;\theta_{1}\;u_{2}\text{,}\;w_{2}\text{,}\;\theta_{2}\right\rfloor }^{T}\text{,}$$

and in the local coordinate system $\left(\overset{¯}{x}\text{,}\;\overset{¯}{z}\right)$ as

(17) $${\overset{¯}{q}}^{e}={\left\lfloor \overset{¯}{u}\text{,}\;{\overset{¯}{\theta }}_{1}\text{,}\;{\overset{¯}{\theta }}_{2}\right\rfloor }^{T}\text{,}$$

where $\overset{¯}{u}$ describes the change of length of the beam, which is related to the axial force $\overset{‾}{N}\text{;}{\overset{¯}{\theta }}_{1}$ (${\overset{¯}{\theta }}_{2}$) denotes the rotation of the axis of the beam at point $\overset{ˆ}{1}$ ($\overset{ˆ}{2}$), which results in the bending moment ${\overset{‾}{M}}_{1}$ (${\overset{‾}{M}}_{2}$). Herein, the upper bar denotes quantities in the local coordinate system $\left(\overset{¯}{x}\text{,}\overset{¯}{z}\right)$.

With the help of the principle of virtual work the matrix ${\overset{∼}{K}}_{T}^{e}$ is obtained as

(18) $${\overset{∼}{K}}_{T}^{e}=X^{T}\cdot {\overset{‾}{K}}_{T}^{e}\cdot X+\frac{z\cdot z^{T}}{\overset{ˆ}{l}}\overset{‾}{N}+\frac{\left(r\cdot z^{T}+z\cdot r^{T})({\overset{‾}{M}}_{1}+{\overset{‾}{M}}_{2}\right)}{{\overset{ˆ}{l}}^{2}}\text{.}$$

In (18), X denotes the matrix for the transformation from local coordinates $\left(\overset{¯}{x}\text{,}\overset{¯}{z}\right)$ to global coordinates (x,z), which is given as

(19) $$X=\left[\begin{array}{cccccc} -\cos\overset{ˆ}{\beta } & -\sin\overset{ˆ}{\beta } & 0 & \cos\overset{ˆ}{\beta } & \sin\overset{ˆ}{\beta } & 0\\ -\sin\overset{ˆ}{\beta }/\overset{ˆ}{l} & \cos\overset{ˆ}{\beta }/\overset{ˆ}{l} & 1 & \sin\overset{ˆ}{\beta }/\overset{ˆ}{l} & -\cos\overset{ˆ}{\beta }/\overset{ˆ}{l} & 0\\ -\sin\overset{ˆ}{\beta }/\overset{ˆ}{l} & \cos\overset{ˆ}{\beta }/\overset{ˆ}{l} & 0 & \sin\overset{ˆ}{\beta }/\overset{ˆ}{l} & -\cos\overset{ˆ}{\beta }/\overset{ˆ}{l} & 1 \end{array}\right]\text{,}$$

where

(20) $$\overset{ˆ}{l}=\sqrt{{\left(x_{2}+u_{2}-x_{1}-u_{1}\right)}^{2}+{\left(z_{2}+w_{2}-z_{1}-w_{1}\right)}^{2}}$$

is the length of the chord of the deformed beam and $\overset{ˆ}{\beta }$ is the angle enclosed by this chord and the *x*-axis, resulting in

(21) $$\cos\overset{ˆ}{\beta }=\frac{x_{2}+u_{2}-x_{1}-u_{1}}{\overset{ˆ}{l}}\text{,}\;\sin\overset{ˆ}{\beta }=\frac{z_{2}+w_{2}-z_{1}-w_{1}}{\overset{ˆ}{l}}\text{.}$$

The vectors r and z were introduced for the sake of a more concise notation. They are given as

(22) $$\begin{array}{cc} & r={\left\lfloor -\cos\overset{ˆ}{\beta }\;-\sin\overset{ˆ}{\beta }\;0\;\cos\overset{ˆ}{\beta }\;\sin\overset{ˆ}{\beta }\;0\right\rfloor }^{T}\text{,}\\ & z={\left\lfloor \sin\overset{ˆ}{\beta }\;-\cos\overset{ˆ}{\beta }\;0\;-\sin\overset{ˆ}{\beta }\;\cos\overset{ˆ}{\beta }\;0\right\rfloor }^{T}\text{;} \end{array}$$

${\overset{¯}{K}}_{T}^{e}$ is the element tangent stiffness matrix in the local coordinate system. Its dimension is 3×3. Differentiation of (18) with respect to *λ* yields

(23) $$\begin{array}{cc} & {\overset{˙}{\overset{∼}{K}}}_{T}\;={\overset{˙}{X}}^{T}\cdot {\overset{‾}{K}}_{T}^{e}\cdot X+X^{T}\cdot {\overset{˙}{\overset{¯}{K}}}_{T}^{e}\cdot X+X^{T}\cdot {\overset{‾}{K}}_{T}^{e}\cdot \overset{˙}{X}+\\ & \frac{(\overset{˙}{z}\cdot z^{T}+z\cdot {\overset{˙}{z}}^{T})\overset{ˆ}{l}-z\cdot z^{T}\overset{˙}{\overset{ˆ}{l}}}{{\overset{ˆ}{l}}^{2}}\overset{‾}{N}+\frac{z\cdot z^{T}}{\overset{ˆ}{l}}\overset{˙}{\overset{‾}{N}}+\\ & \frac{(\overset{˙}{r}\cdot z^{T}+r\cdot {\overset{˙}{z}}^{T}+\overset{˙}{z}\cdot r^{T}+z\cdot {\overset{˙}{r}}^{T}){\overset{ˆ}{l}}^{2}}{{\overset{ˆ}{l}}^{2}}({\overset{‾}{M}}_{1}+{\overset{‾}{M}}_{2})-\\ & \frac{2(r\cdot z^{T}+z\cdot r^{T})\overset{˙}{\overset{ˆ}{l}}}{{\overset{ˆ}{l}}^{3}}({\overset{‾}{M}}_{1}+{\overset{‾}{M}}_{2})+\\ & \frac{r\cdot z^{T}+z\cdot r^{T}}{{\overset{ˆ}{l}}^{2}}({\overset{˙}{\overset{‾}{M}}}_{1}+{\overset{˙}{\overset{‾}{M}}}_{2})\text{,} \end{array}$$

where

(24) $$\begin{array}{cc} & \overset{˙}{\overset{ˆ}{l}}=r^{T}\cdot {\overset{˙}{q}}^{e}\text{,}\;\overset{˙}{\overset{ˆ}{\beta }}=\frac{z^{T}\cdot {\overset{˙}{q}}^{e}}{\overset{ˆ}{l}}\text{,}\\ & \overset{˙}{r}=\frac{z^{T}\cdot z}{\overset{ˆ}{l}}{\overset{˙}{q}}^{e}\text{,}\;\overset{˙}{z}=-\frac{r^{T}\cdot z}{\overset{ˆ}{l}}{\overset{˙}{q}}^{e}\text{,} \end{array}$$

(25) $$\overset{˙}{X}=\left[\begin{array}{cccccc} x_{11} & x_{12} & 0 & -x_{11} & -x_{12} & 0\\ x_{21} & x_{22} & 0 & -x_{21} & -x_{22} & 0\\ x_{21} & x_{22} & 0 & -x_{21} & -x_{22} & 0 \end{array}\right]\text{,}$$

with

(26) $$\begin{array}{cc} & x_{11}=\sin\overset{ˆ}{\beta }\overset{˙}{\overset{ˆ}{\beta }}\text{,}\;x_{12}=-\cos\overset{ˆ}{\beta }\overset{˙}{\overset{ˆ}{\beta }}\text{,}\\ & x_{21}=-\frac{\cos\overset{ˆ}{\beta }\overset{˙}{\overset{ˆ}{\beta }}\overset{ˆ}{l}-\sin\overset{ˆ}{\beta }\overset{˙}{\overset{ˆ}{l}}}{{\overset{ˆ}{l}}^{2}}\text{,}\;x_{22}=-\frac{\sin\overset{ˆ}{\beta }\overset{˙}{\overset{ˆ}{\beta }}\overset{ˆ}{l}+\cos\overset{ˆ}{\beta }\overset{˙}{\overset{ˆ}{l}}}{{\overset{ˆ}{l}}^{2}}\text{,} \end{array}$$

and

(27) $${\overset{˙}{q}}^{e}=A^{e}\cdot \overset{˙}{q}\text{,}$$

with $\overset{˙}{q}$ denoting the vector of nodal displacement rates.

In (18), (23), $X\text{,}\;\overset{˙}{X}\text{,}\;\overset{ˆ}{l}\text{,}\;\overset{˙}{\overset{ˆ}{l}}\text{,}\;\overset{ˆ}{\beta }\text{,}\;\overset{˙}{\overset{ˆ}{\beta }}\text{,}\;r\text{,}\;\overset{˙}{r}\text{,}\;z$, and $\overset{˙}{z}$ are purely geometric quantities. They are independent of the beam theory used for derivation of the expressions for $\overset{‾}{N}\text{,}\;{\overset{‾}{M}}_{1}\text{,}\;{\overset{‾}{M}}_{2}$, and ${\overset{¯}{K}}_{T}^{e}$ and of their derivatives with respect to *λ*.

For the Euler–Bernoulli theory, the axial displacement *u* and the transverse deflection *w* are given as

(28) $$u=\frac{\overset{¯}{x}}{l}\overset{¯}{u}\text{,}$$

(29) $$w=\overset{¯}{x}\left(1-\frac{\overset{¯}{x}}{l}\right){\overset{¯}{\theta }}_{1}+\frac{{\overset{¯}{x}}^{2}}{l}\left(\frac{\overset{¯}{x}}{l}-1\right){\overset{¯}{\theta }}_{2}\text{.}$$

With the help of (28), (29), ${\overset{¯}{K}}_{T}^{e}$ is obtained as

(30) $${\overset{¯}{K}}_{T}^{e}=\left[\begin{array}{ccc} \frac{\mathrm{EA}}{l} & 0 & 0\\ 0 & \frac{4{\mathrm{EI}}_{y}}{l} & \frac{2{\mathrm{EI}}_{y}}{l}\\ 0 & \frac{2{\mathrm{EI}}_{y}}{l} & \frac{4{\mathrm{EI}}_{y}}{l} \end{array}\right]$$

where *A* denotes the area of the cross-section of the beam. Herein it is assumed to be constant along the axis of this beam. $I_{y}$ is the moment of inertia about the $\overset{¯}{y}$-axis. The vector of internal forces ${\overset{¯}{F}}^{e}={\left\lfloor \overset{‾}{N}\text{,}\;{\overset{‾}{M}}_{1}\text{,}\;{\overset{‾}{M}}_{1}\right\rfloor }^{T}$ is given as

(31) $${\overset{¯}{F}}^{e}={\overset{¯}{K}}_{T}^{e}\cdot {\overset{¯}{q}}^{e}=\left[\begin{array}{c} \frac{\mathrm{EA}}{l}\overset{¯}{u}\\ \frac{4{\mathrm{EI}}_{y}}{l}{\overset{¯}{\theta }}_{1}+\frac{2{\mathrm{EI}}_{y}}{l}{\overset{¯}{\theta }}_{2}\\ \frac{2{\mathrm{EI}}_{y}}{l}{\overset{¯}{\theta }}_{1}+\frac{4{\mathrm{EI}}_{y}}{l}{\overset{¯}{\theta }}_{2} \end{array}\right]\text{.}$$

Differentiation of (30), (31) with respect to *λ*, assuming the cross-section and the material properties to be constant, yields

(32) $${\overset{˙}{\overset{¯}{K}}}_{T}^{e}=0$$

and

(33) $${\overset{˙}{\overset{¯}{F}}}^{e}={\overset{¯}{K}}_{T}^{e}\cdot {\overset{˙}{\overset{¯}{q}}}^{e}={\overset{¯}{K}}_{T}^{e}\cdot (X\cdot {\overset{˙}{q}}^{e})\text{.}$$

Thus, all quantities appearing in (23) are known. All of them are functions of either $q^{e}$ or ${\overset{˙}{q}}^{e}$.

## 4 Finite difference approximations of ${\overset{˙}{\overset{∼}{K}}}_{T}$

The approach described in Section 3 depends on the chosen element. Therefore, it is impractical in view of the great number of types of finite elements and the large variety of technical problems, requiring the choice of one or more problem-specific elements.

Alternatively, ${\overset{˙}{\overset{∼}{K}}}_{T}$ can be approximated by means of the finite difference method [13], [14]. Based on two different definitions of the first derivative of ${\overset{∼}{K}}_{T}$ with respect to *λ*, two alternative finite-difference approximations of ${\overset{˙}{\overset{∼}{K}}}_{T}$ are considered:

### 4.1 Load-based finite-difference approximation of ${\overset{˙}{\overset{∼}{K}}}_{T}$

${\overset{˙}{\overset{∼}{K}}}_{T}$ is approximated by a forward two-point finite-difference expression, i.e.

(34) $${\overset{˙}{\overset{∼}{K}}}_{T}\approx \frac{{\overset{∼}{K}}_{T}(\lambda +h)-{\overset{∼}{K}}_{T}(\lambda )}{h}\text{,}$$

where *h* denotes a small change of *λ*, which is positive for loading and negative for unloading.

### 4.2 Displacement-based finite-difference approximation of ${\overset{˙}{\overset{∼}{K}}}_{T}$

${\overset{˙}{\overset{∼}{K}}}_{T}$, which was already defined in (2), is redefined as a directional derivative [6], i.e.

(35) $${\overset{˙}{\overset{∼}{K}}}_{T}≔K_{T\text{,}q}\cdot \overset{˙}{q}={\left(\frac{d}{\mathrm{dh}}\right|}_{h=0}K_{T}(q+h\overset{˙}{q})\text{,}$$

where *h* is a small positive number. Following from (35), ${\overset{˙}{\overset{∼}{K}}}_{T}$ can be approximated by a forward two-point finite-difference expression, i.e.

(36) $${\overset{˙}{\overset{∼}{K}}}_{T}\approx \frac{K_{T}(q+h\overset{˙}{q})-{\overset{∼}{K}}_{T}(q)}{h}\text{,}$$

where $K_{T}(q+h\overset{˙}{q})$, contrary to ${\overset{∼}{K}}_{T}(q)$, does not refer to points located on primary equilibrium paths.

**Fig. 3:**
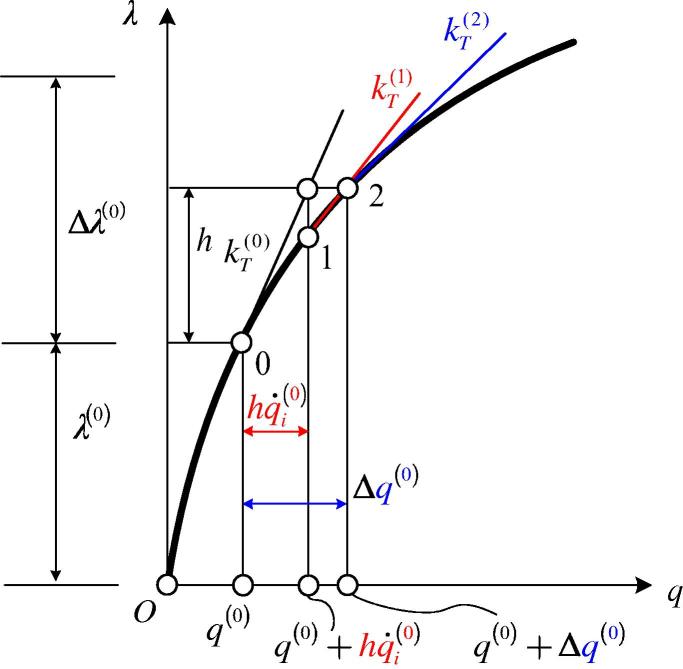
Comparison of two alternative finite-difference approximations of ${\overset{˙}{k}}_{T}(\lambda^{\left(0\right)})≔{\overset{˙}{k}}_{T}^{\left(0\right)}$.

Fig. 3 refers to a comparison of the two alternative finite-difference approximations of ${\overset{˙}{\overset{∼}{K}}}_{T}$ for the special case of a system with one DOF. For this special case, (34) is replaced by

(37) $${\overset{˙}{k}}_{T}^{\left(0\right)}\approx \frac{k_{T}^{\left(2\right)}-k_{T}^{\left(0\right)}}{h}\text{,}$$

where $k_{T}^{\left(0\right)}$ and $k_{T}^{\left(2\right)}$ denote the slopes of the tangents to the curve λ(q) at point 0 and point 2, respectively, and $h⩽\Delta \lambda^{\left(0\right)}$. Computation of $k_{T}^{\left(2\right)}$ requires an iteration.

Alternatively, (36) is replaced by

(38) $${\overset{˙}{k}}_{T}^{\left(0\right)}\approx \frac{k_{T}^{\left(1\right)}-k_{T}^{\left(0\right)}}{h}\text{.}$$

In contrast to computation of $k_{T}^{\left(2\right)}$, computation of $k_{T}^{\left(1\right)}$ does not require an iteration. This was one of the reasons for implementing the algorithm for determination of the DBFDA of ${\overset{˙}{\overset{∼}{K}}}_{T}$ into the commercial FE program MSC.MARC 2012 [15]. Moreover, as follows on closer inspection of Fig. 3, the right-hand side of (38) is a better approximation of ${\overset{˙}{k}}_{T}^{\left(0\right)}$ than the right-hand side of (37). Only if *h* is so small that the test of convergence of the iteration is satisfied already after the first iteration step, the two approaches are equivalent.

The analytical expression and the finite difference approximations of ${\overset{˙}{\overset{∼}{K}}}_{T}$ were implemented in FEMv2, which is a Matlab-based nonlinear finite element program.

## 5 Numerical examples

### 5.1 Circular arch

**Fig. 4:**
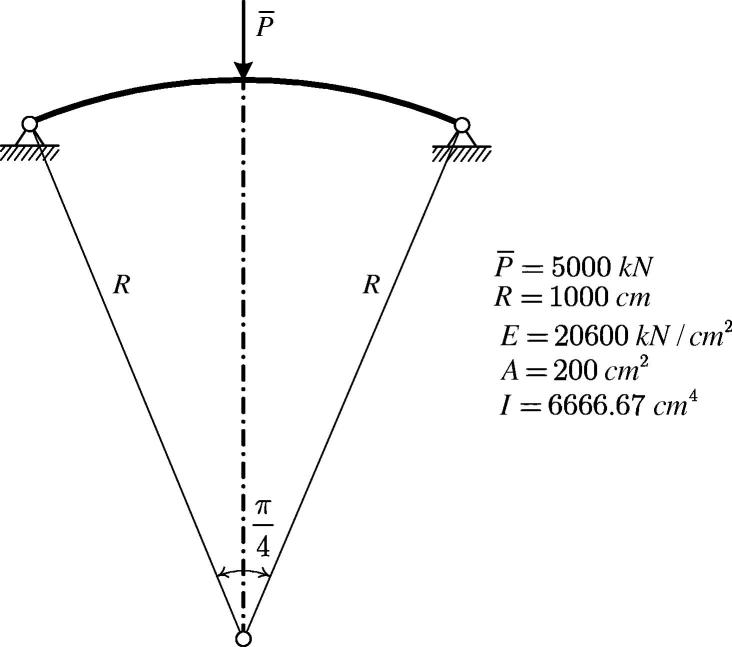
Configuration and material properties of a circular arch subjected to a vertical point load.

Fig. 4 shows a circular arch subjected to a vertical point load $\overset{‾}{P}$ at the vertex. The geometric properties and the material parameters are also shown in this figure. The arch is discretized by 100 two-node beam elements. It is analyzed by means of FEMv2 as well as by MSC.MARC 2012.

**Fig. 5:**
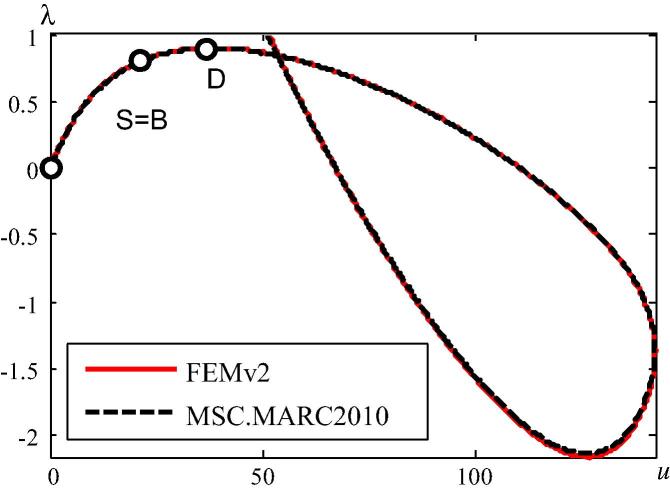
Load–displacement paths.

The vertical displacement of the central node is chosen as the representative degree of freedom. The load–displacement paths obtained from the two FE codes are shown in Fig. 5. The result obtained from MSC.MARC 2012 agrees very well with the one obtained from FEMv2. S=B denotes a bifurcation point which, in the given case, is the stability limit. Hence, the snap-through point *D* has no physical significance.

**Fig. 6:**
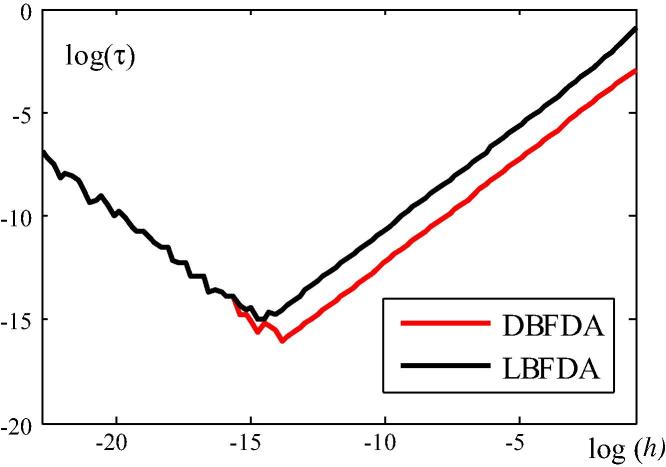
log(h)–log(τ) diagram for error assessment of the norm of ${\overset{˙}{\overset{∼}{K}}}_{T}$, based on the DBFDA and the LBFDA, respectively, of this matrix.

**Fig. 7:**
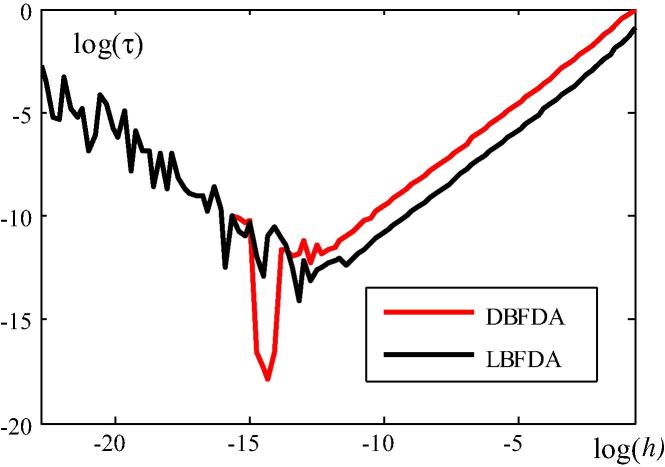
log(h)–log(τ) diagram for error assessment of $\lambda_{1}^{*}$, based on the DBFDA and the LBFDA, respectively, of this quantity.

The quality of the DBFDA and LBFDA of ${\overset{˙}{\overset{∼}{K}}}_{T}$ is assessed by comparing the dependence of a suitable error norm of ${\overset{˙}{\overset{∼}{K}}}_{T}$ (see Fig. 6) and of the error of $\lambda_{1}^{*}$ (see Fig. 7) on *h*. In Fig. 6,

(39) $$\tau ≔\frac{\left\|{\overset{˙}{\overset{∼}{K}}}_{T}{\_}_{\mathrm{DBFDA}(\mathrm{LBFDA})}-{\overset{˙}{\overset{∼}{K}}}_{T}{\_}_{\mathrm{EX}}\right\|}{\left\|{\overset{˙}{\overset{∼}{K}}}_{T}{\_}_{\mathrm{EX}}\right\|}\text{.}$$

where ${\overset{˙}{\overset{∼}{K}}}_{T}{\_}_{\mathrm{EX}}$ indicates calculation of ${\overset{˙}{\overset{∼}{K}}}_{T}$ from the analytical expression derived in Section 2, and ${\overset{˙}{\overset{∼}{K}}}_{T}{\_}_{\mathrm{DBFDA}(\mathrm{LBFDA})}$ refers to its calculation as a DBFDA and LBFDA, respectively. The norm of the two matrices in (39) is defined as follows:

(40) $$\left\|C\right\|≔\frac{\sum_{i=1}^{n}\sum_{j=1}^{n}c_{\mathrm{ij}}^{2}}{n^{2}}$$

where $c_{\mathrm{ij}}$ is the coefficient in the *i*th row and the *j*th column of C . The red (black) curve illustrates the dependence of the error norm of ${\overset{˙}{\overset{∼}{K}}}_{T}$, based on the DBFDA (LBFDA) of this matrix, on *h*. For log(h)>∼-15, the error of the LBDFA is larger than the one of the DBFDA, which corroborates a statement in connection with the explanation of Fig. 3.

The convergence rate of a numerical approach for calculation of a quantity is directly related to the slope of the curve in a *log–log* plot [16]. The red line in Fig. 6 is parallel to the black line, indicating that the convergence rate of the error norm based on the DBFDA of ${\overset{˙}{\overset{∼}{K}}}_{T}$ is the same as the one of the error norm based on the LBFDA of this matrix. For log(h)<∼-14, the error is increasing, which is the consequence of the limitation of the number of digits in the representation of numbers in the computing machine. Hence, the lowest point of each curve characterizes the accuracy of the respective FDA. According to Fig. 6, the accuracy of the DBFDA is higher than the one of the LBFDA.

The second quantity used for assessing the accuracy of the two finite difference approximations is $\lambda_{1}^{*}$. The results of this assessment are shown in Fig. 7. Herein,

(41) $$\tau ≔\frac{\left|\lambda_{1}^{*}{\_}_{\mathrm{DBFD}(\mathrm{LBFD})}-\lambda_{1}^{*}{\_}_{\mathrm{EX}}\right|}{\left|\lambda_{1}^{*}{\_}_{\mathrm{DBFD}}(h=0.5)-\lambda_{1}^{*}{\_}_{\mathrm{EX}}\right|}\text{,}$$

where $\lambda_{1}^{*}{\_}_{\mathrm{EX}}$ indicates calculation of $\lambda_{1}^{*}$ from the analytical solution, and $\lambda_{1}^{*}{\_}_{\mathrm{DBFDA}(\mathrm{LBFDA})}$ refers to calculation of $\lambda_{1}^{*}$ from the DBFDA and the LBFDA, respectively, of $\lambda_{1}^{*}$.

For log(h)>∼-13, the error of the DBDFA is larger than the one of the LBFDA. This can be explained by investigating the special case of a system with one DOF (see Fig. 3). Solving the CLE for this special case yields

(42) $$\lambda_{1-\mathrm{DBFDA}}^{*(0)}=-\frac{k_{T}^{\left(0\right)}}{{\overset{˙}{k}}_{T-\mathrm{DBFDA}}^{\left(0\right)}}+\lambda^{\left(0\right)}$$

and

(43) $$\lambda_{1-\mathrm{LBFDA}}^{*(0)}=-\frac{k_{T}^{\left(0\right)}}{{\overset{˙}{k}}_{T-\mathrm{LBFDA}}^{\left(0\right)}}+\lambda^{\left(0\right)}\text{,}$$

respectively, where ${\overset{˙}{k}}_{T-\mathrm{DBFDA}}^{\left(0\right)}$ (${\overset{˙}{k}}_{T-\mathrm{LBFDA}}^{\left(0\right)}$) denotes the DBFDA (LBFDA) of ${\overset{˙}{k}}_{T}^{\left(0\right)}$. Since ${\overset{˙}{k}}_{T-\mathrm{LBFDA}}^{\left(0\right)}<{\overset{˙}{k}}_{T-\mathrm{DBFDA}}^{\left(0\right)}<0$ for the same value of *h*, which follows from Fig. 3,

(44) $$\lambda_{1-\mathrm{DBFDA}}^{*(0)}>\lambda_{1-\mathrm{LBFDA}}^{*(0)}\text{.}$$

as follows from (42), (43). (44) indicates that the error of $\lambda_{1}^{*(0)}$, resulting from the DBFDA of ${\overset{˙}{k}}_{T}^{\left(0\right)}$, is larger than that based on the LBFDA.

Nevertheless, the DBFDA of $\lambda_{1}^{*}$ has the same convergence rate as the LBFDA. For the same error tolerance within the accuracy range of each approximation, $t_{\mathrm{DBFDA}}<t_{\mathrm{LBFDA}}$, where *t* stands for the computer time. E.g., for $\tau_{\mathrm{tol}}=10^{-10}\text{,}t_{\mathrm{DBFDA}}=16s<72s=t_{\mathrm{LBFDA}}$. Hence, concerning computing time, the DBFDA of $\lambda_{1}^{*}$ is superior to the LBFDA of this quantity, which overcompensate the slightly larger error of the former for relevant values of *h*.

**Fig. 8:**
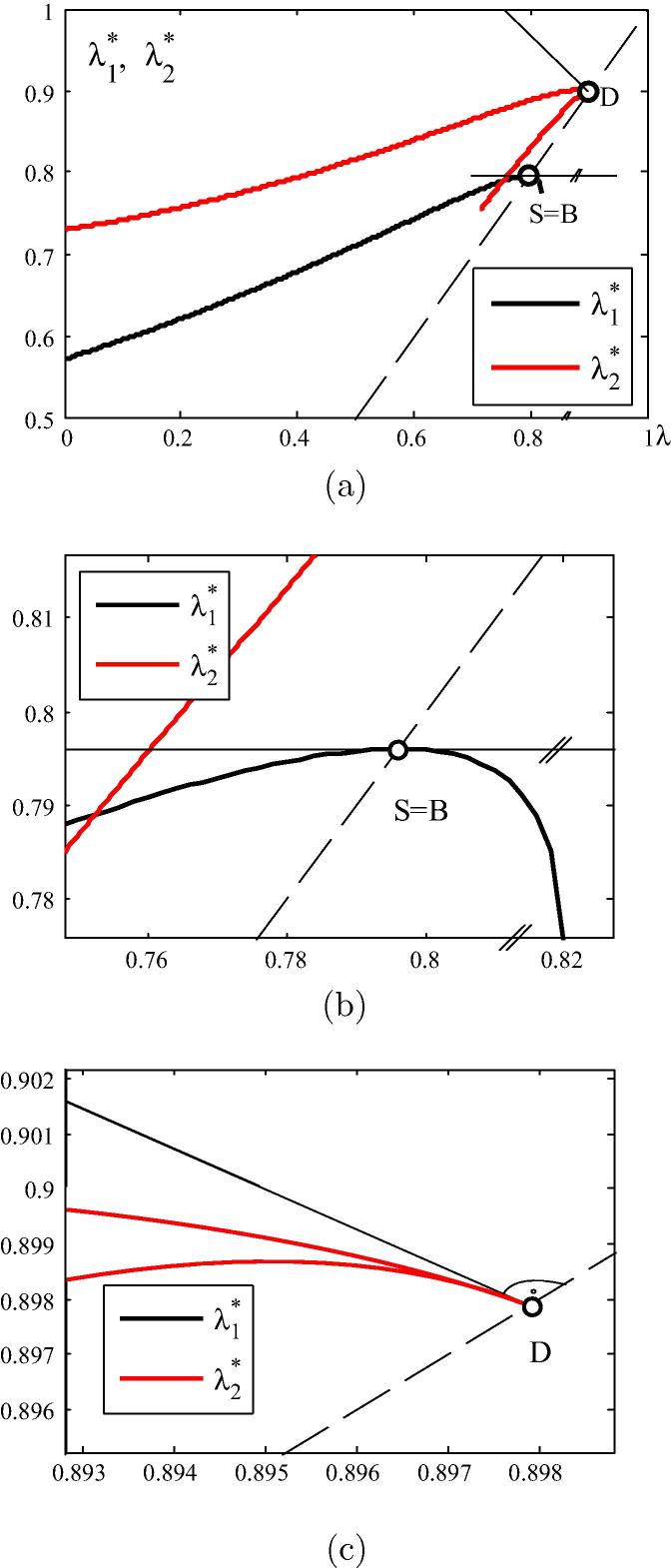
$\lambda_{1}^{*}$-*λ* and $\lambda_{2}^{*}$-*λ* diagram (a) full scale, (b) detail nearby point *B*, (c) detail nearby point *D*.

Fig. 8(a) shows the $\lambda_{1}^{*}$-*λ* and the $\lambda_{2}^{*}$-*λ* diagram, related to the first two eigenvalues of the CLE. The $\lambda_{1}^{*}$-*λ* curve has a horizontal tangent at the bifurcation point *B* (see Fig. 8(b)), whereas the $\lambda_{2}^{*}$-*λ* curve has a cusp of second kind, with a tangent normal to the diagonal, i.e. to the dashed line in Fig. 8(a) and (c).

**Fig. 9:**
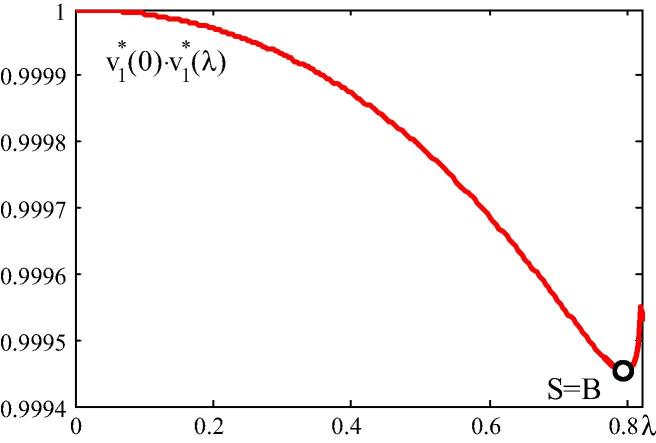
$v_{1}^{*}(\lambda =0)\cdot v_{1}^{*}(\lambda )$ diagram.

In Fig. 9, the product of $v_{1}^{*}(\lambda =0)\cdot v_{1}^{*}(\lambda )$, which is related to the angle enclosed by the vectors $v_{1}^{*}(\lambda )$ and $v_{1}^{*}(\lambda =0)$, is plotted. The curve has a minimum at *B*, which indicates that the prebuckling state involves both membrane and bending stresses.

### 5.2 Thrust-line arch

Fig. 5 shows a two-hinged arch subjected to a vertical uniform load. The geometric form of the axis of the arch is given as

(45) $$x\in [0\text{,}L]\;y=\frac{4h}{L^{2}}x(l-x)\text{.}$$

**Fig. 10:**
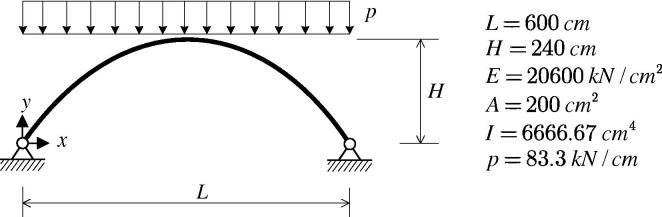
Configuration and material properties of a two-hinged arch subjected to a vertical uniform load.

Geometric parameters and material data are shown in Fig. 10. Contrary to a three-hinged arch of the same geometric configuration, subjected to the same load as the two-hinged arch shown in Fig. 10, the latter, strictly speaking, is no thrust-line arch. However, since its bending moments are negligibly small [14], it is justified to speak of a thrust-line arch. For such an arch, buckling occurs from a membrane stress state.

**Fig. 11:**
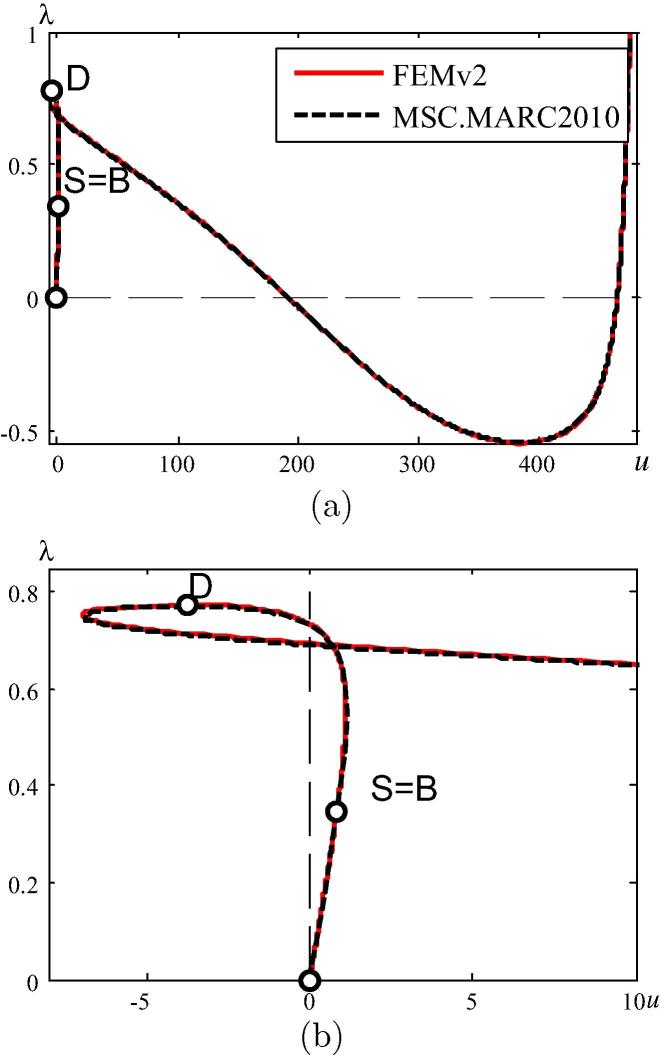
Load–displacement paths (a) whole curves, (b) initial part of curves.

Herein, the structure is analyzed by means of FEMv2 as well as MSC.MARC 2012. The arch is discretized by 100 two-node beam elements. The vertical displacement of the central node is chosen as the representative degree of freedom. The load–displacement paths obtained from FEMv2 and MSC.MARC 2012 are shown in Fig. 11. The results obtained from MSC.MARC 2012 agree very well with the ones obtained from FEMv2. Fig. 11(a) shows the entire computed load–displacement curve, containing a bifurcation point S=B, followed by a snap-through point, *D*. Hence, the latter is physically insignificant. Fig. 11(b) illustrates the initial part of the load–displacement path.

**Fig. 12:**
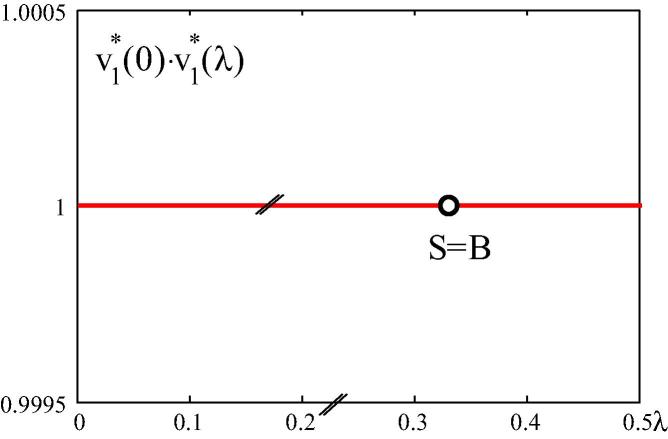
$v_{1}^{*}(\lambda =0)\cdot v_{1}^{*}(\lambda )$ diagram.

Fig. 12 shows a plot of the function $v_{1}^{*}(\lambda =0)\cdot v_{1}^{*}(\lambda )$ for the thrust-line arch subjected to a uniformly distributed load. The straight line indicates that $v_{1}^{*}$ is constant in the prebuckling regime, which confirms a remarkable mathematical property of the CLE, meaning that $v_{1}^{*}$ is constant for a membrane stress state in the prebuckling regime.

## 6 Conclusions

Motivated by visualization of the concept of energy-based categorization of buckling problems by means of spherical geometry, methods for numerical solutions of the CLE, representing the mathematical tool for this categorization, were proposed and evaluated in this paper.

• The two-dimensional co-rotational beam element, used in FEMv2, was found to be suitable for static structural stability analysis. The structural response agrees very well with the one obtained by means of the commercial finite element program MSC.MARC 2012.
• The DBFDA of ${\overset{˙}{\overset{∼}{K}}}_{T}$ is more accurate than the LBFDA of this matrix.
• The convergence rate of the DBFDA of ${\overset{˙}{\overset{∼}{K}}}_{T}$ and of the numerical solution for $\lambda_{1}^{*}$ based on this approximation is the same as that of the LBFDA of ${\overset{˙}{\overset{∼}{K}}}_{T}$ and of the numerical solution for $\lambda_{1}^{*}$ based on this approximation.
• The numerical solution for $\lambda_{1}^{*}$ based on the DBFDA of ${\overset{˙}{\overset{∼}{K}}}_{T}$ is less accurate than the one based on the LBFDA of this matrix. However, for the same error tolerance, the former is superior to the latter as regards computing time.
• The eigenvector $v_{1}^{*}$ for the circular arch subjected to a vertical point load at the vertex changes its direction in the prebuckling regime, which reflects the existence of bending. The angle enclosed by $v_{1}^{*}(\lambda )$ and $v_{1}^{*}(\lambda =0)$ becomes a maximum at the stability limit.
• The eigenvector $v_{1}^{*}$ for the thrust-line arch subjected to a vertical uniform load is constant, which indicates that there is no bending before buckling.

The present work paves the way for the implementation of a stable and accurate numerical approach for the solution of the CLE in MSC.MARC 2012, which is a necessary prerequisite for the numerical realization of a new concept of categorization of buckling problems with the help of spherical geometry.
